# A jejunal gastrointestinal stromal tumor with massive gastrointestinal hemorrhage treated by emergency surgery: A case report

**DOI:** 10.1097/MD.0000000000030098

**Published:** 2022-09-02

**Authors:** Beata Jabłońska, Paweł Szmigiel, Piotr Wosiewicz, Jan Baron, Weronika Szczęsny-Karczewska, Sławomir Mrowiec

**Affiliations:** a Department of Digestive Tract Surgery, Medical University of Silesia, Katowice, Poland; b Department of Gastroenterology and Hepatology, Medical University of Silesia, Katowice, Poland; c Department of Radiology, Medical University of Silesia, Katowice, Poland; d Department of Pathomorphology and Molecular Diagnostics, Faculty of Medical Science, Medical University of Silesia, Katowice, Poland.

**Keywords:** COVID-19, gastrointestinal bleeding, gastrointestinal stromal tumor

## Abstract

A jejunal gastrointestinal stromal tumor (GIST) is a rare neoplasm of the gastrointestinal (GI) tract. Massive bleeding due to a jejunal GIST is a diagnostic and therapeutic challenge. It may be a life-threatening GIST complication that requires urgent intervention. Acute GI bleeding, which requires urgent surgical intervention, is a very rare clinical manifestation of GIST. A jejunal GIST with massive hemorrhage with coronavirus disease 2019 in a male patient in older age with many comorbidities has been not reported in the worldwide literature.

**Methods:**

In this case report, we present an 80-year-old man who was admitted to surgery due to abdominal pain, melena, and hematochezia for several hours. An upper endoscopy and colonoscopy were inconclusive. A multidetector contrast-enhanced computed tomography (CT) of the abdominal and pelvic cavity showed concentric irregular thickening in the distal jejunum.

The histopathological finding showed a GIST measuring 6 cm with a mitotic index 2/50 high power fields. The patient’s hemodynamic condition deteriorated despite initial conservative treatment including a blood transfusion. Therefore, patient underwent the emergency surgery 24 hours after admission: partial jejunal resection with the tumor followed by primary end-to-end anastomosis.

**Results:**

The mass was removed completely. There were no surgical complications in the postoperative course. On the first postoperative day, a severe acute respiratory syndrome coronavirus 2 polymerase chain reaction test was performed due to a persistent dry cough, which yielded a positive result. After 14 days, the patient died due to pneumonia and circulatory failure.

**Conclusions:**

This case indicates that jejunal GIST can present as massive lower gastrointestinal bleeding and urgent surgery can successfully stop bleeding and save the patient’s life. The CT scan was the most effective investigation to find the source of GI bleeding in this case. Therefore, we suggest performing CT in patients with acute massive lower gastrointestinal bleeding when the source of bleeding is not visible on endoscopy, and urgent surgical jejunal resection to stop life-threatening bleeding caused by a jejunal GIST.

## 1. Introduction

Lower gastrointestinal bleeding (LGIB) is a frequent reason for hospitalization especially in the elderly.^[1]^ Gastrointestinal stromal tumors (GISTs) are rare tumors, with an estimated incidence of around 1/100,000/year.^[2]^ They are the most common mesenchymal neoplasms of the gastrointestinal (GI) tract.^[2–5]^

GISTs, first described by Mazur and Clark in 1983, originate from the interstitial cells of Cajal.^[6]^ These tumors can arise anywhere in the GI tract, but their most frequent locations are the stomach (50%) and small intestine (25%).^[4]^ Jejunal GISTs are extremely rare (0.1%–3% of all GI tumors).^[5]^

Coronavirus disease 2019 (COVID-19) is caused by the severe acute respiratory syndrome coronavirus 2 and has caused a worldwide pandemic.^[7]^ Association between comorbidity (including cardiovascular disorders, hypertension, chronic obstructive pulmonary disease, chronic kidney disease; other: liver disease, GI disorders, immunocompromised, neurological disorders, psychiatric disorders, metabolic disorders, blood disorders, transplant, chronic pancreatitis, connective tissue disorder, smoking, obesity, hyperlipidemia) and COVID-19 fatality was assessed; and 3 out of 4 studies showed a strong correlation between having 1 or more comorbidity and disease fatality. Also, a higher age (>60) is a risk factor for severe COVID-19 outcome.^[8]^

We report the first case of GIST of the small intestine with acute massive GI bleeding treated by emergency, with coexisting COVID-19.

## 2. Case report

An 80-year-old man was admitted to surgery due to abdominal pain, melena, and hematochezia for several hours. His medical history consisted of myocardiac infarction, chronic heart failure, multiple coronary stents implantation, arterial hypertension, and chronic obstructive pulmonary disease.

**Figure 1.**
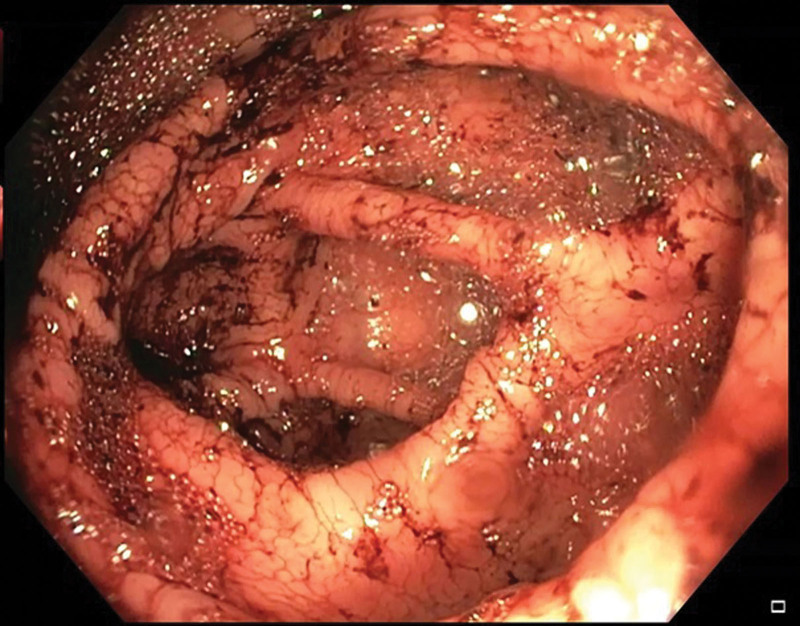
A colonoscopy showing the cecum with blood present in the intestine despite the fact that full preparation for colonoscopy with polyethylene glycol was carried out in accordance with the protocol.

**Figure 2.**
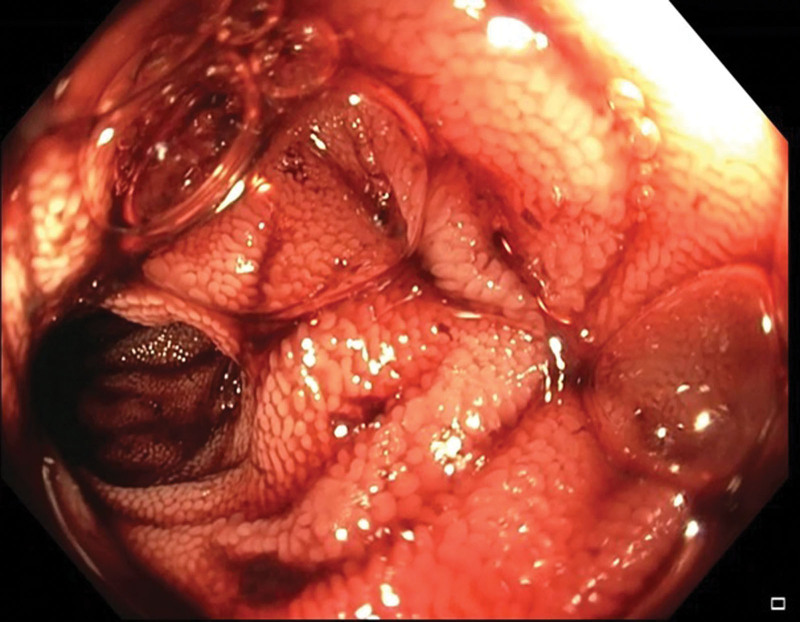
A colonoscopy showing the ileum with significant blood content.

**Figure 3.**
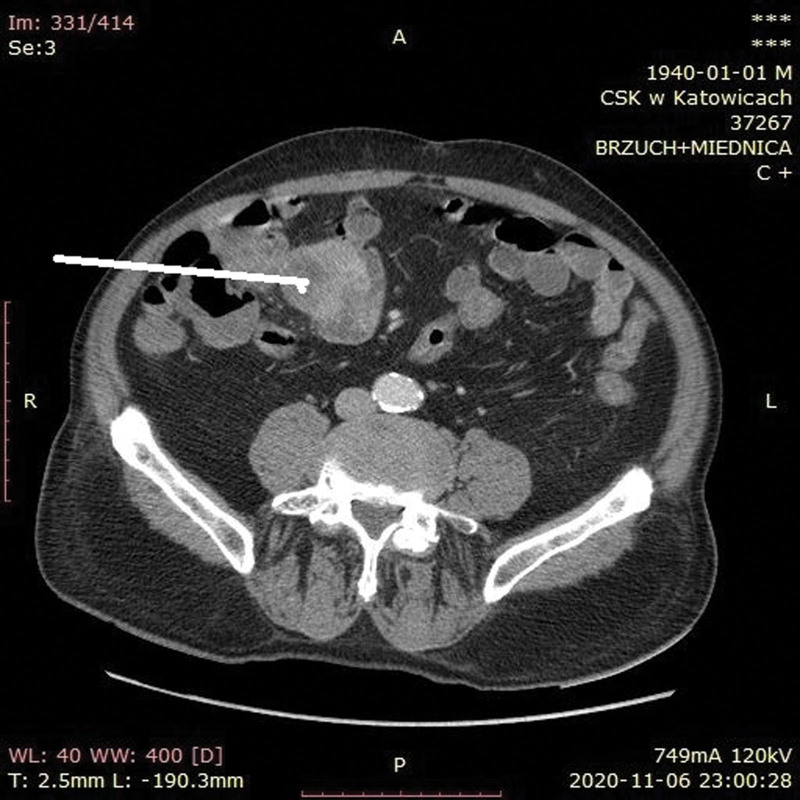
A multidetector contrast-enhanced CT of the abdominal and pelvic cavity showing concentric irregular thickening in the distal jejunum (white arrow).

On admission, the patient was hemodynamically stable (blood pressure 110/50 mm Hg, heart rate 72/min). His initial hemoglobin level was 7.2 g/dL (reference range, 13.5–16.5 g/dL), red blood cell (RBC) count was 2.39 × 10^6^/μL (reference range, 4.2–5.7 × 10^6^/μL), white blood count was 4.51 × 10^3^/μL (reference range, 4.0–10.0 × 10^3^/μL), platelet count was 126 × 10^3^/μL (reference range, 130–400 × 10^3^/μL), hematocrit was 22.0% (reference range, 40%–53%), activated partial thromboplastin time was 32.2 seconds (reference range, 25.4–36.9 seconds), prothrombin time was 12.2 seconds (reference range, 9.4–12.5 seconds), international normalized ratio was 1.08 (reference range, 0.80–1.20), and prothrombin activity was 90% (reference range, 90%–120%). Immediately, 3 units of RBCs were transfused. Urgent upper endoscopy showed no GI pathology. A colonoscopy revealed hematic residues and no bleeding source (Figs. 1 and 2). The patient’s condition deteriorated with hemoglobin levels decreased to 5.8 mg/dL. Consecutive transfusion of RBC (9 units) and fresh frozen plasma (4 units) was performed. An estimated summarized blood loss was approximately 3000 mL. After endoscopic investigations, a tumor located within small intestine was suspected. Therefore, a multidetector contrast-enhanced computed tomography (CECT) of the abdominal and pelvic cavity was performed. The examination was performed after oral administration of a 3% iodinated positive contrast solution and intravenous administration of 85 ml of Omnipaque 350 in order to evaluate the bowel tumor. CECT showed concentric irregular thickening in the distal jejunum with high point areas within the rebuilt wall densities (that might correspond to local bleeding) in the arterial phase (Fig. 3). We decided to perform CECT (not angio-CT) due to suspicion of the intestinal tumor as the source of LGIB. Our goal was to simultaneously visualize the bowel tumor and bleeding. Angio-CT would not show it exactly.

**Figure 4.**
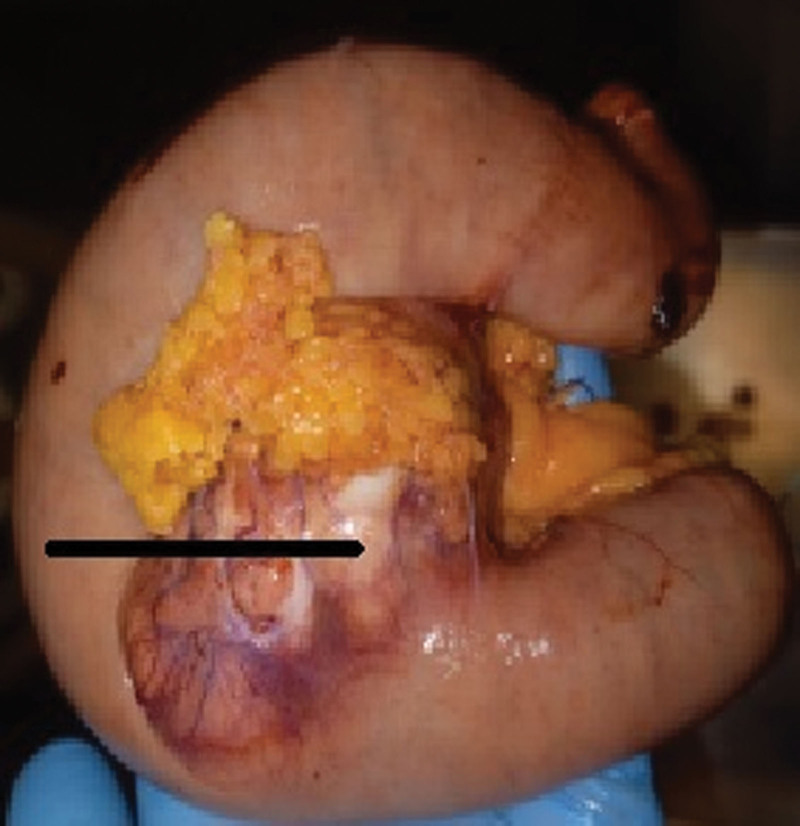
A resected segment of the jejunum with the tumor (black arrow). CT = computed tomography.

Due to the patient’s unstable hemodynamic condition, an emergency laparotomy after 24 hours from admission was performed. Intraoperatively, in the jejunum (approximately 120 cm distally to the Treitz’s ligament), the tumor of diameter of about 5 cm in adhesions with greater omentum was found. Partial resection of the jejunum including the tumor, followed by primary end-to-end jejuno-jejunal anastomosis, was performed (Fig. 4). After the intestine was cut during resection, the small intestine filled with blood was shown. There were no surgical complications and no recurrent GI bleeding in the postoperative course. On the first postoperative day, a severe acute respiratory syndrome coronavirus 2 reverse transcriptase polymerase chain reaction test was performed due to a persistent dry cough, which yielded a positive result. The patient was not screened for COVID-19 at admission (there was no hospital COVID-19 screening program at that moment). On the 7th postoperative day, the patient was transferred to the Internal Medicine Unit for the treatment of COVID-19 pneumonia with no surgical problems. After 14 days, the patient died due to pneumonia and circulatory failure.

**Figure 5.**
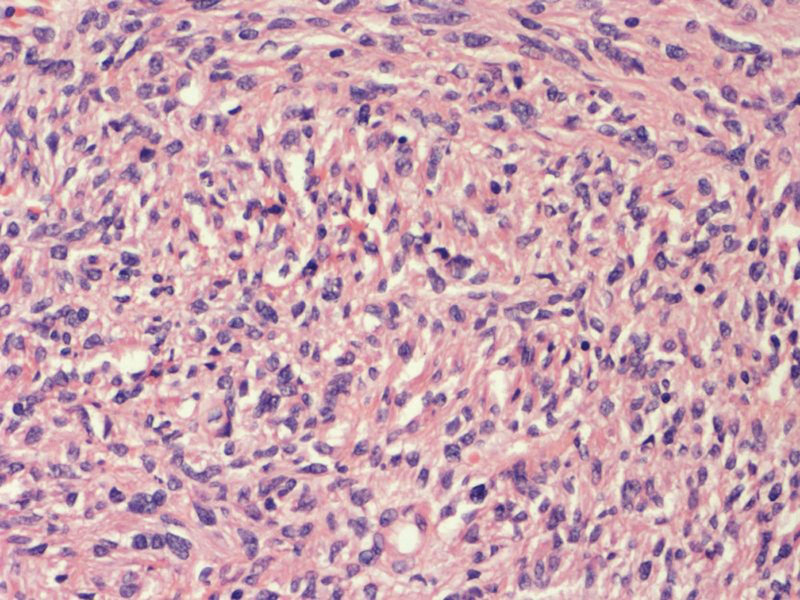
Histopathological findings: gastrointestinal stromal tumor (hematoxylin and eosin staining, magnification ×20).

**Figure 6.**
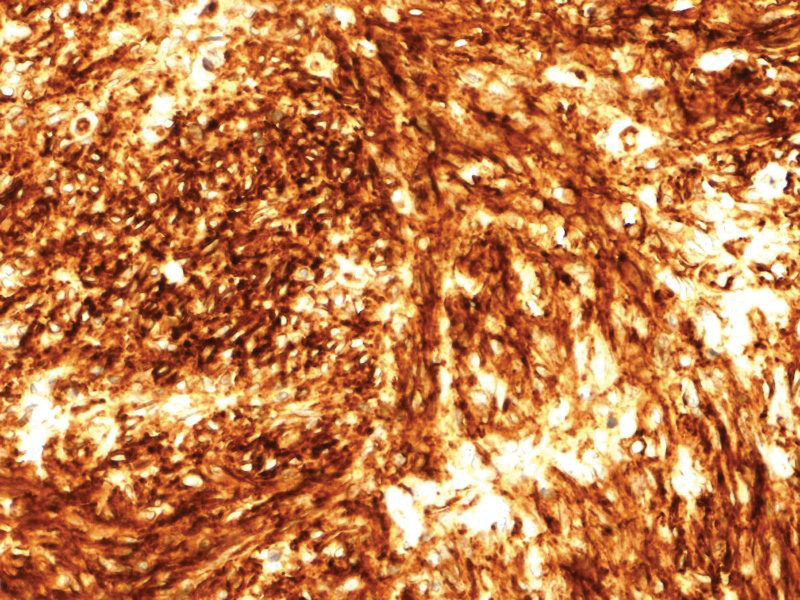
Histopathological findings: immunohistochemical staining in tumor cells: CD34(+) (magnification ×20).

**Figure 7.**
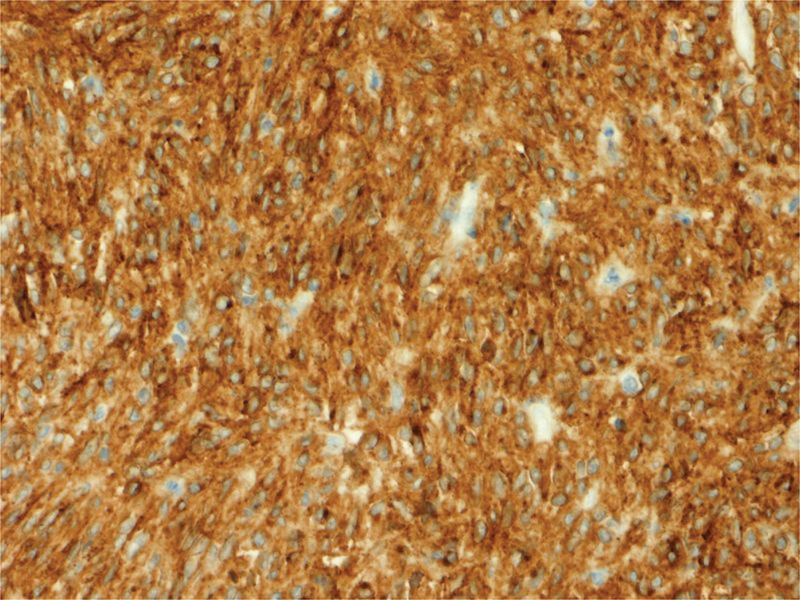
Histopathological findings: immunohistochemical staining in tumor cells: CD117(+) (magnification ×20).

**Figure 8.**
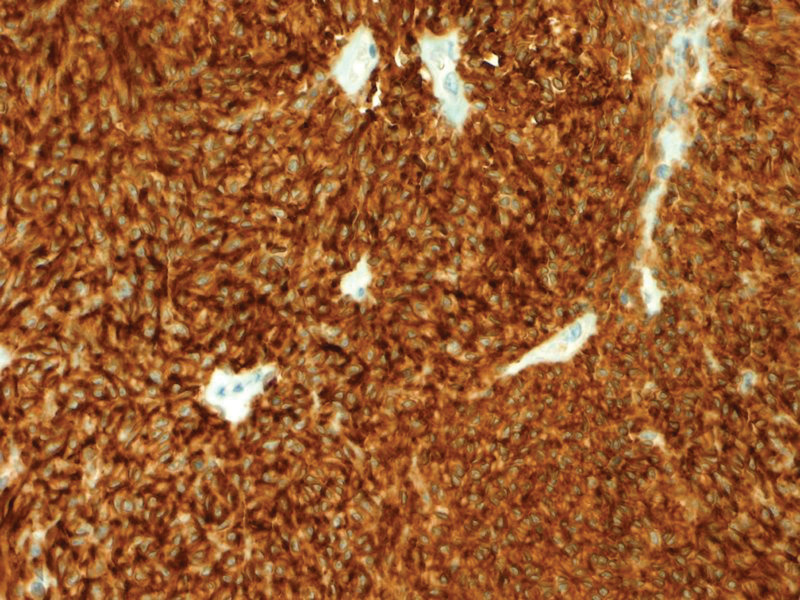
Histopathological findings: immunohistochemical staining in tumor cells: DOG(+) (magnification ×20).

The histopathological finding showed a GIST measuring 6 cm with a mitotic index 2/50 high power fields involving the jejunal wall from submucosa to the serosa. The focal neoplastic tissue infiltrated the periintestinal adipose tissue. There was a necrotized area within the tumor with extensive hemorrhage. Minor foci of necrosis were present in the remaining tissue of the tumor. There were no features of angioinvasion. There was no neoplastic tissue within the proximal, distal, and radial resection margins. Therefore, the surgical margins were clear. The intermediate risk for malignancy was reported and 1 metastatic lymph node in the periintestinal adipose tissue was found. The medium risk of aggressive behavior was recognized according to National Institute of Health consensus criteria for GIST risk according to Fletcher et al^[9,10]^ and T3N1 staging according to the American Joint Committee on Cancer Staging Manual (8^th^ edition) Tumor Node Metastasis classification,^[11]^ respectively. Immunohistochemistry revealed CD34(+), CD117(+), DOG(+), S-100 (−), and SMA (−). Histopathological findings including hematoxylin and eosin staining as well as immunohistochemical staining are presented in Figures 5–8.

## 3. Discussion

In 20% of patients, GISTs are asymptomatic. The clinical symptoms of GISTs depend on tumor size, location, and others. GI bleeding and abdominal discomfort are common clinical signs. The incidence of clinical symptoms is the following: GI bleeding (30%–40%), abdominal pain (20%–50%), and obstruction (10%–30%).^[12]^

GI bleeding is the most dangerous GIST complication. Anemia, emaciation, and melena are typical signs in patients with chronic GI bleeding. In cases of acute bleeding, the manifestation may include hemorrhagic shock and peritonitis.^[12]^

Prognostic factors in patients with GISTs are the mitotic index, tumor size, tumor location (gastric GISTs have a better prognosis compared to small bowel or rectal GISTs), and tumor rupture.^[2]^

Tumors larger than 4 cm can lead to a life-threatening GI hemorrhage caused by overlying mucosa with ulceration and necrosis, perforation, and obstruction. According to the literature, GI bleeding caused by GISTs may be related to mucosal and submucosal destruction by tumor growth, invasion of nutrient vessels leading to vascular rupture, tumor necrosis, and the joint action of digestive juices, GI peristalsis, and fecal transmission.^[12]^ According to a recent meta-analysis by Fan et al,^[13]^ the location of GIST in the small intestine, tumor diameter ≥ 5 cm, mitotic index ≥ 5/50 high power field, and tumor rupture increase the risk of GI bleeding in patients with GIST.^[13]^ In this patient, the intestinal location and large tumor diameter (6 cm) were the risk factor of GI bleeding.

Although intermittent GI bleeding is the most common manifestation of a GIST, massive life-threatening GI bleeding requiring urgent treatment (surgery, endoscopic treatment, or arterial embolization) is an extremely rare presentation of GIST.^[5,12,14–23]^ Several reports regarding jejunal GISTs with acute GI bleeding have been reported in the literature.^[5,14–23]^ Our patient presented massive LGIB. Histopathological finding confirmed tumor necrosis and secondary focal hemorrhagic foci.

We present a jejunal GIST with acute massive LGIB requiring an immediate diagnosis. CT, not endoscopy, revealed the LGIB source. Upper and lower GI endoscopy are the first line investigation of GI bleeding. Small intestinal bleeding is a diagnostic challenge as this region is inaccessible by conventional endoscopy. The imaging modalities required after negative upper and lower GI endoscopy include capsule endoscopy, CT angiography, conventional CT scan with intravenous +/− oral contrast, double balloon enteroscopy, and magnetic resonance enterography.^[19,22]^ Because of hemodynamic instability, after inconclusive endoscopic investigations, conventional CT scan with intravenous and oral contrast was performed at night in our patient. CT allowed the quick and accurate diagnosis.

The hemodynamically unstable patient underwent successful emergency surgery involving partial jejunal resection with primary end-to-end anastomosis. According to Current European Society for Medical Oncology-EURACAN Clinical Practice Guidelines for diagnosis, treatment, and follow-up, the standard treatment of localized GISTs is complete surgical excision of the lesion, with no dissection of clinically negative lymph nodes in order to achieve R0 resection.^[2]^ According to the literature, surgical resection and radiologic embolization are the most effective methods in the management of GISTs with acute bleeding.^[23–25]^ Therefore, in this case, urgent surgical treatment stopped LGIB and was oncologically radical.

In this patient, despite emergency successful radical surgery saving a life, a fatal outcome was caused by COVID-19 coexisting with risk factors such as high age and many comorbidities (cardiovascular disorders, hypertension, chronic obstructive pulmonary disease).

## 4. Conclusion

We suggest performing CT in patients with acute massive LGIB when the source of bleeding is not visible on endoscopy, and urgent surgical jejunal resection to stop life-threatening bleeding caused by a jejunal GIST.

## Author contributions

**Conceptualization:** Beata Jabłońska.

**Data curation:** Beata Jabłońska, Jan Baron, Paweł Szmigiel, Piotr Wosiewicz.

**Formal analysis:** Beata Jabłońska.

**Investigation:** Beata Jabłońska, Jan Baron, Paweł Szmigiel, Piotr Wosiewicz, Weronika Szczęsny-Karczewska.

**Methodology:** Beata Jabłońska, Paweł Szmigiel.

**Supervision:** Sławomir Mrowiec.

**Visualization:** Weronika Szczęsny-Karczewska.

**Writing – original draft:** Beata Jabłońska, Paweł Szmigiel.

**Writing – review & editing:** Beata Jabłońska.
